# Paying for treatments? Influences on negotiating clinical need and decision-making for dental implant treatment

**DOI:** 10.1186/1472-6963-9-7

**Published:** 2009-01-12

**Authors:** Catherine E Exley, Nikki S Rousseau, Jimmy Steele, Tracy Finch, James Field, Cam Donaldson, J Mark Thomason, Carl R May, Janice S Ellis

**Affiliations:** 1 Institute of Health and Society, 21 Claremont Place, Newcastle University, Newcastle upon Tyne, NE2 4AA, UK; 2 School of Dental Sciences, Framlington Place, Newcastle University, Newcastle upon Tyne, NE2 4BW, UK

## Abstract

**Background:**

The aim of this study is to examine how clinicians and patients negotiate clinical need and treatment decisions within a context of finite resources. Dental implant treatment is an effective treatment for missing teeth, but is only available via the NHS in some specific clinical circumstances. The majority of people who receive this treatment therefore pay privately, often at substantial cost to themselves. People are used to paying towards dental treatment costs. However, dental implant treatment is much more expensive than existing treatments – such as removable dentures. We know very little about how dentists make decisions about whether to offer such treatments, or what patients consider when deciding whether or not to pay for them.

**Methods/Design:**

Mixed methods will be employed to provide insight and understanding into how clinical need is determined, and what influences people's decision making processes when deciding whether or not to pursue a dental implant treatment. Phase 1 will use a structured scoping questionnaire with all the General dental practitioners (GDPs) in three Primary Care Trust areas (n = 300) to provide base-line data about existing practice in relation to dental implant treatment, and to provide data to develop a systematic sampling procedure for Phase 2. Phases 2 (GDPs) and 3 (patients) use qualitative focused one to one interviews with a sample of these practitioners (up to 30) and their patients (up to 60) to examine their views and experiences of decision making in relation to dental implant treatment. Purposive sampling for phases 2 and 3 will be carried out to ensure participants represent a range of socio-economic circumstances, and choices made.

**Discussion:**

Most dental implant treatment is conducted in primary care. Very little information was available prior to this study about the quantity and type of treatment carried out privately. It became apparent during phase 2 that ISOD treatment was an unusual treatment in primary care. We thus extended our sample criteria for Phase 3 to include people who had had other implant supported restorations, although not single tooth replacements.

## Background

In the UK, and other western societies, people are living longer lives with less acute infectious disease, but experiencing more long term degenerative conditions in later life. As a result, medical care is increasingly being focussed on managing chronic illness, rather than curative treatment; improving and maintaining quality of life is a key concern for health care provision. Further, ongoing medical and technological advances offer increasing possibilities for maintenance of health and quality of life. Although recent political debate has emphasised the need for the increased use of technologies in the delivery of health care services[1,2], this must be considered within a context of a growing proportion of the population that might benefit from such treatments. This presents a problem for those responsible for delivering health care in a context of finite resources, and decisions must be made about how such resources should be allocated [3]. In health care systems, like the NHS, which are based on the premise that health care should be free at the point of delivery, identifying ways of balancing the supply and demand of finite resources is becoming increasingly necessary [4].

One mechanism of addressing finite resources is for patients to pay a contribution towards the cost of their treatment. Within this particular model of providing and financing health care it is useful to understand how clinical need and treatment decisions are negotiated. This is necessary for three reasons: Firstly, any rationing of services impacts on the everyday practice of health care professionals who must make decisions about the provision of services to individual patients using criteria which are beyond perceived clinical need, thereby according greater significance to non-clinical influences on decision-making [5]. Secondly, patients themselves are faced with additional choices and decisions to make about treatments and services offered to them. They not only need to consider their perceived clinical need, but also any potential benefits to their quality of life, as well as assessing the value of such benefits if they as individual patients are required to make a part or whole contribution. Thirdly, resource rationing, and the increasing consideration required of non-medical decision making factors, has important implications for how *clinical need *itself gets negotiated and reconfigured through the process of decision-making.

This study will use the provision of complete implant-supported overdentures (ISOD) as the vehicle to develop generically applicable insights into the processes of negotiating clinical need and making treatment decisions within a context of rationed resources. Implants are devices that can be placed directly into the bone of the jaws to replace lost teeth and/or provide support for dentures. They are highly effective as they stabilize the denture and allow much improved function. ISODs have been shown to improve patient satisfaction and edentulous patients' oral related quality of life in this clinical situation which can be regarded as a chronic disability or handicap [6-8]. The physical impairment brought about by the loss of natural teeth represents a chronic disability because it negatively impacts on daily living activities, such as eating and speaking. A proportion of denture wearers may also be considered to be handicapped as they will actually avoid performing such activities in front of anyone outside close family members [9,10]. ISODs are currently only available without charge through referral to secondary care. However, this option is only available to a very small proportion of these patients with very specific clinical needs. Within primary care dentistry, however, individuals are increasingly being able to choose implant treatment for which they incur a personal financial cost. Therefore this provides an excellent clinical setting which is constrained by finite resources in which to examine the process of patients and clinicians negotiating clinical need and treatment decisions.

Current evidence suggests that most edentulous patients would benefit from ISODs, and this form of treatment has been proposed by the McGill Consensus as the minimal standard of care [8], however, there are not enough resources available to enable this. Within the current context there are two main groups who qualify for the provision of ISOD without incurring a personal financial cost within secondary care. The first is patients with severe denture intolerance including severe resorption of the lower jaw. These patients would normally be referred from their GDPs or from within the hospital. The second group comprises patients with apparently satisfactory anatomy but who find it more difficult than most to adapt to the limitations of complete dentures. Although it would be expected that secondary care provides implant treatment for those with the most marked problems, this is not necessarily the case. Those who receive such treatments may do so because of there being no available provision of ISODs within primary care in their locality, the referral patterns of their GDP or because they are more persistent in seeking a solution for their problems. Similarly, within primary care personal clinical experience suggests that people from a range of socio-economic backgrounds are willing to pay for ISOD.

Whilst the barriers to providing ISODs are not clear, they are likely to include the fact that, although an increasing number of GDPs have training and experience to carry out implant procedures, only a minority choose to do so. Compounding this issue is the fact that people now maintain natural teeth much longer, with the resultant effect being that the age at which people may become edentulous is rising. People in older age are likely to find adapting to dentures even more difficult and thus experience more problems which would be ameliorated by implants. Thus, the disparity between resources and demand for treatment services is increasing, and therefore a greater understanding of the processes of decision-making around treatment such as these that have personal cost implications, from the perspectives of both patients and dentists is required.

Finite health care resources have implications for practitioners and patients and also for how clinical need is determined and negotiated. Practitioners draw on a range of 'non-medical' factors in making decisions about how to treat their patients [5,11]. Broadly, such factors have been characterised as relating to characteristics of the patient; characteristics of the clinician; and features of the practice setting[5]. Demographic, socio-economic and social factors are known to be important in influencing treatment decisions made by health professionals. However, it has not yet been investigated how professionals' approaches to treatment decision-making may be affected by situations where the treatment option may incur personal financial costs for the patient. Limited existing research suggests that professionals themselves may adopt various strategies, including concentrating their discussions about possible treatments on those who are perceived to be able to afford to pay [3]. Given the link between low-income and ill-health, this is unlikely to be a strategy which will meet all patient need and, indeed, may increase inequalities in health and access to health services[3]. Questions also exist concerning how professionals make judgements and decisions about referrals to scarce secondary care resources, and what treatment options and choices to offer to which patients. In the context of dentistry, the day to day decisions made by GDPs have a significant influence on the oral health of the population [12]. Whilst much previous research has concentrated on the influence of clinical factors on treatment decisions, it is increasingly acknowledged that factors such as the financial environment and patients' preferences might be expected to exert a major influence on dentists' decision making practices but are, as yet, poorly understood [13,14]. In relation to ISODs, limited work in the area suggests that the relative influence of a range of factors (oral, medical and personal) on decision-making appears to vary greatly between different practitioner groups [15]; this requires further investigation. Understanding the process by which health professionals, including dentists, make treatment decisions is becoming increasingly relevant in the context of increasing expectations of evidence based practice. Not only is it essential for dentists to consider the patient's values for alternative treatments and outcomes [16] but it is also argued that dentists should combine the patient's treatment needs and preferences with the best available scientific evidence in conjunction with their own clinical expertise [17,18].

As well as considering the implications for clinicians it is also important to note that the involvement and participation of patients, and the public generally, in decision-making about health care is a key priority in health policy [19,20], which has attracted a substantive body of empirical research [21]. Patients, and their roles in decision-making, have been conceptualised in various ways ranging from 'self-managers' [22] to an increasing emphasis on the patient as a 'customer' [23]. In reality however, such roles for patients are rarely realised [24] and indeed patients may choose to adopt different 'roles' at different points within and without the clinical encounter. Clearly, in a health provision context where demand for services is increasing and resources are limited, patients themselves will be increasingly required to make decisions about treatments that involve additional personal costs to themselves, and which might bring considerable improvement to their quality of life and/or self esteem. Patient participation in decisions about dental treatments is beginning to attract empirical attention. Initial findings indicate that dental patients do have distinct preferences in relation to decision-making roles, and that these may not always be met in their interactions with their dentists [25].

When making treatment decisions clinicians and patients must first determine clinical need. It is well recognised that social factors are particularly pertinent to practitioner-patient consultations [26]. In dentistry, it is acknowledged that economic constraints may present problems for the definition and assessment of clinical need [27]. It is argued that the assessment of need, and decision-making processes, should be considered on the three levels of the patient, dentists, and society. A key challenge for decision making around need in the context of dentistry is to establish the legitimate roles of these various parties, so that the concept of need can be used as a basis for planning and provision of services. Such goals would be advanced through a better understanding of how clinical need is defined and assessed by both patients and practitioners in decisions concerning treatments that have personal cost implications.

The benefits of a clearer understanding of negotiating clinical need and treatment decisions within the context of limited and finite resources is of interest to patients, purchasers and providers of health care services. At a national level, strategic planning and implementation of the delivery of a National Health Service is dependant on a full understanding of issues such as the uptake of care for the management of chronic conditions from the private sector. Those who commission the delivery of state funded health and social care (through Primary Care and Social Care Trusts), will need to devise strategies for delivering health services that reflect an understanding of decision making priorities and willingness to pay on the part of those who use them. Service providers (practitioners) themselves need to understand the complex, hitherto unexplored relationship between payment, effectiveness of treatment and demand in a health service which is increasingly a mix of public and privately funding. Those who insure patients against the costs of private treatment also have an interest in the process of decision making where a financial commitment is required.

This multi-disciplinary study will provide generically applicable and timely insights into an increasingly pertinent area of how clinical need and treatment decisions are negotiated within a context of finite resources. It will address this issue by examining in detail how, in a health care system with finite resources, clinical need and treatment decisions are mediated by perceived costs: physical, social, psychological and financial. Further, by addressing this neglected area of clinical research of interactions within dental consultations, it will develop new insights and understandings of the decision-making process within dentistry.

## Aims and objectives

The aim of this study is to examine how clinicians and patients negotiate clinical need and treatment decisions within a context of finite resources.

This study will develop understandings of how both health professionals and patients negotiate clinical need and make decisions about treatment options. In particular it will examine how interpretations of need and subsequent decisions about treatment are mediated by social and psychological factors, as well as the financial environment in which such joint decision making takes place. Specifically it will:

1. Examine how notions of clinical need are negotiated by both patients and health professionals,

2. Critically investigate the relationship between constructs of clinical need and treatment decisions,

3. Assess the relative influences of professional, physicality (symptoms and/or aesthetics), social and psychological factors on patients' decision making processes, and

4. Compare and contrast the ways in which patients' decision making processes may change when they incur a personal financial cost.

## Methods

In order to begin to understand this under-researched area this project will take an inductive approach to examine in detail both practitioners' and patients' experiences. Mixed methods will be employed to provide insight and understanding into how clinical need is determined, and what influences people's decision making processes when deciding to pursue a particular course of treatment. Phase 1 will use a structured scoping questionnaire to provide base-line data about existing practice. Phases 2 and 3 will both use qualitative focused interviews with practitioners and patients to examine their views and experiences. Focused interviews are a particularly useful tool to employ in an area where relatively little is known about the ways in which people make decisions. They are flexible enough to allow interviewer and interviewee to explore issues which are pertinent to the individual person which had not been anticipated in advance, thus enabling a fuller understanding of the processes at work to emerge. The study comprises three inter-related phases:

### Phase 1 – Establishing existing practice

The aims of Phase 1 are:

• To provide accurate data about the proportion of GDPs offering implants as a treatment option

• To provide data to develop a systematic sampling procedure for Phase 2

There are two potential treatments available to edentulous patients: conventional dentures and ISOD. In order to establish the relative influences on negotiating clinical need and pursuing a particular treatment option, it is necessary to identify those GDPs who do offer patients the possibility of implant over dentures. Presently there are no data providing us with this information. *Sample: *All primary care dental practices across the North East England Region (Newcastle upon Tyne, North Tyneside and Northumbria) (n = 135 practices; 300 GDPs). *Method: *As no existing questionnaire is available, a postal questionnaire will be designed and pre-tested with a panel of dental practitioners. An introductory letter will be sent to all GDPs explaining the nature of the research. This will be followed 2 weeks later by a postal questionnaire with instructions and a stamped addressed envelope for the return of the questionnaire. Included with this will be an information sheet relating to phases 2 and 3 and a form asking for the practitioners consent to be contacted with a view to further participation in the research. One reminder will be sent four weeks after initial posting. *Analysis: *Data will be entered into a suitable software package e.g. SPSS and simple descriptive statistical analysis will be conducted. The data from these questionnaires will identify four groups of GDPs: 1) those who offer implant treatment themselves (for which patients must pay); 2) those who refer to other colleagues within primary care or secondary care (for which patients must pay); 3) those who to refer to secondary care where a limited supply of implants are available free of charge to patients with particular clinical needs; 4) those who do not offer implants – conventional dentures only. These data serve two purposes: 1) to describe current practice within the region; 2) to provide the sampling frames for Phases 2 & 3 (see table 1 below).

**Table 1:** Sampling and methods summary:

**Phase 1: Screening questionnaire – All GDPs to identify:**						
Personally provide implant treatment		Refer on				Never offer
		Refer within primary care		Refer to Secondary		

**Phase 2: Interviews with GDPs about practice**						

Personally provide implant treatment		Refer within primary care		Refer to Secondary		
To provide retrospective and prospective sample for Phase3						

**Phase 3: Interviews with patients retrospectively and prospectively**						

Offered treatment by another GDP		Offered treatment by own GDP		Referred to secondary care		
Accept Implants	Refuse Implants	Accept Implants	Refuse Implants	Accept Implants	Refuse Implants	

### Phase 2 – Practitioners' experiences

The aims of Phase 2 are:

• To determine how practitioners establish clinical need for implants

• To determine the perceived physical, social, psychological, financial factors which may affect whether implant treatments is offered

• To determine from practitioners' accounts how implant treatment is offered to patients

• To determine practitioners' views about why people choose particular treatments

*Sampling*: The sample (up to 30) will include three groups of GDPs: 1) those who offer implant treatment with a cost to the patient within primary care; 2) those who refer to others who offer implant treatment with a cost to the patient within primary care 3) those who refer to secondary care with no cost to the patient (see table 1 below). A purposive sample strategy will be used in order to ensure that the full breadth of socio-economic practice settings (rural: urban, deprived: affluent) is represented in the sample. *Method: *Focused interviews will be conducted on an individual basis with GDPs. Written informed consent will be obtained at the time of but prior to the interview. All interviews will be audio-recorded. Clinicians will be paid for their time to take part in the interview.

### Phase 3: Patients' experiences

*The aims of Phase 3 are*:

• To gather patients' accounts of how treatment options were presented to them

• To examine patients' accounts of their care throughout their treatment

• To determine the relative influence of social, psychological, physical and financial factors on their treatment decision

• To compare the experiences of those who incurred a personal financial cost with those who did not

*Sampling*: Patients (up to 60) who have been *offered *implant treatment in different settings will be recruited via their practitioners in Phase 2 (or, where necessary, via secondary care). It is anticipated that there may be a relatively small number of people going through implant treatment at any one time, and coupled with issues of recruiting for research studies, patient recruitment will take place both retrospectively and prospectively. The sample will include people who have very recently been offered implant treatment as well as identifying those who were offered treatment up to 12 months ago. We will recruit patients who have chosen to pay for ISOD in primary care, those who refused ISOD in primary care, and those who were offered ISOD treatment, which incurred no personal financial cost, in secondary care. This will identify 6 different groups of patients who have been offered and either accepted or refused treatment by: their own GDP, another GDP, or secondary care (see table 1). A purposive sample strategy will be used in order to ensure that the full breadth of socio-economic situations is represented in the sample. Patients will be invited to opt-in to the study. Although it is likely that patients and practitioners will be recruited from the same practices, for ethical and methodological reasons it is not intended that these data will be linked in any way for the purpose of questioning or analysis. This will allow us to preserve respondents' anonymity thus encouraging open and honest responses as necessary to examine the relative influences of different factors on people's decisions to pursue (or not) a particular treatment. *Method: *As with Phase 2, focused interviews will be conducted on a one-to-one basis with patients in a location convenient to them. GDPs from Phase 2 will be asked to identify patients who fulfil the inclusion criteria and to send these people an information pack about the study. This information pack will contain a study information sheet, a consent to contact form and a reply paid envelope for the return of the form to the study research team. This design means that no patient information will need to be released by the dentist to the study research team. No reminders will be used. Patients who choose to return a consent to contact form will be contacted by a study researcher and given an opportunity to further discuss their potential involvement in a study interview. If the patient verbally agrees to participate in an interview, patients will be given a choice of venue and offered an interview time that suits them. Written informed consent will be obtained at the time of but prior to the patient interview.

#### Data Preparation and Analysis for Phases 2 and 3

All interviews will be digitally recorded and transcribed verbatim. In line with Data Protection Legislation and Research Governance all information pertaining to individuals will be anonymised. Data collection and analysis will occur concurrently to allow for issues which arise in earlier interviews in a particular phase of the study to be explored in more depth in subsequent interviews in that phase. Thematic analysis, based on the 'constant comparative method' [28,29] will be employed. The validity of data interpretation will be ensured by independent coding and cross-checking by at least two members of the research team. A suitable software package (e.g. NVivo) will be used to facilitate the management of data analysis. The study team includes social scientists (CE, NR); dentists (JS, JE, JF, MT); psychologists (TF) and health economists (CD). Regular team meetings and data sessions throughout the data collection and analysis period will ensure we maximise the benefit to the study of these diverse perspectives.

#### Conceptual analytic framework

In order to deliver the objectives of the study, in the analysis of data from Phases 2 & 3 the following conceptual framework will be applied to ascertain how decisions about clinical need and treatment decisions are mediated by different social, psychological, clinical and financial factors. Each of these concepts relates directly to the objectives of the study and will form the basis for a robust conceptual model.

1. Affect – how do issues of self-perception and self-esteem affect how clinical need is negotiated, and which treatment decisions are made? (Objectives 1,2)

2. Conceptualisation – how does the lived experience affect decision making (e.g. functionality, quality of life) affect how clinical need is negotiated, and which treatment decisions are made? (Objectives 1,2)

3. Life-world – how do personal relationships (e.g. family, friends), socioeconomic circumstances, and interactions within the patient's life world affect the decision making process? (Objectives 3,4)

4. Clinical world – how do interactions within the clinical setting (e.g. Practitioner advice; knowledge of treatment; availability of treatment) affect decisions? (Objectives 3,4)

### Ethical approval

This study has approval from Sunderland NHS Local Research Ethics Committee (Ref:06/Q0904/25), and NHS R&D approval from each participating site. Those in direct contact with patients have been issued with honorary NHS contracts. The study has been the subject of an NHS R&D research governance audit.

There are no particular ethical problems with this study. The main issue relates to maintaining confidentiality of participant data, which will be collected and held in accordance with the Data Protection Act, 1998, and the requirements of NHS research governance. Recruitment of patients will be carried out by their dentists on an "opt-in" basis. Care will be taken to ensure that individual participants are not identifiable in any published outputs from the study.

## Discussion

Prior to this study, there was a lack of information available about the scope of dental implant treatment in the UK. Thus we designed this study on the basis of limited information and some of our preconceptions proved different from the situation found in practice. Three differences that impacted on phase 3 are discussed here:

1. It became apparent after the study started that ISOD treatment was unusual in primary care, at least in our study area; most patients with missing teeth who paid for private treatment paid for a fixed restoration. We therefore extended our sampling criteria for phase 3 to include patients with other types of implant supported restoration, although we continued to exclude patients who had considered a straight forward single tooth replacement.

2. We also found that, because secondary care (NHS) dental implant treatment is only "offered" to a small number of people with severe problems with denture tolerance, that there were very few people who turned down this treatment if offered. This meant that the numbers of people available to recruit to the study (the "refuse implants" box in the secondary care column of table 1) was very small. Findings from a previous study[30] suggested that some patients will turn down the opportunity for dental implant treatment even when there is no cost to themselves. However that study was carried out in the context of a trial which included people who were not experiencing significant problems with their existing conventional dentures. This represents a very different patient population to those seen in UK secondary care.

3. Implicit in the design of the study was the idea that most patients would be offered implant treatment by their usual GDP who would either perform the treatment themselves or refer the patient to another GDP. In practice, we have found a high level of self referral by patients to implant providers.

## Conclusion

Both the dental consultation and private health care represent neglected areas of research. This study offers the opportunity to develop an empirical, theoretical and practical understanding of the dynamics of treatment resources, patient preferences and decision making processes for treatments which incur a personal financial cost. It will have wider general applicability to an increasing number of other areas of service provision where patients may be required to make a decision about paying for treatments. The study will provide information about the likely demand for health care treatments and services which incur a personal cost to patients and the trade-offs patients are prepared to make in order to achieve their desired outcome when they are faced with the fact that they have to make a personal financial contribution. This study also has relevance to the broader debate on tackling health inequalities by examining the extent to which mediating factors such as patient payment distort the alignment of health care provision with clinical need.

## Competing interests

CE, NR, JS, TF, CD. JF, CRM have no competing interests to declare. MT and JE have previously received research funding, and speaker's honoraria from companies with an interest in dental implant treatments

## Authors' contributions

CE, JS, TF, CD, MT, CRM, JE were all involved in original conception and design of the study. JF was involved in the design and conduct of data collection for phase 1. NR was involved in design and conduct of data collection for phases 2 and 3. NR drafted this manuscript. All authors were involved in revising the manuscript critically for important intellectual content and have given approval of the final manuscript.

## Pre-publication history

The pre-publication history for this paper can be accessed here:


